# Quantitative Assessment of Tensile Strength and Degradation Coefficient of m-Aramid/p-Aramid Blended Yarns Used for Outer Layers of Firefighter Clothing under Ultraviolet Light and Correlation with Fabrics Data

**DOI:** 10.3390/polym14193948

**Published:** 2022-09-21

**Authors:** Kaoru Wakatsuki, Souta Onoda, Minami Matsubara, Norimichi Watanabe, Limin Bao, Hideaki Morikawa

**Affiliations:** 1 Institute of Fiber Engineering, Shinshu University, Ueda 386-8567, Nagano, Japan; 2 Faculty of Textile Science and Technology, Shinshu University, Ueda 386-8567, Nagano, Japan

**Keywords:** ultra violet, tensile strength, high-performance fiber, aramid, degradation, curve fitting, ageing

## Abstract

The quantitative relationship between the fraction of UV exposure energy and the retention fraction of tensile strength was investigated on the m-Aramid/p-Aramid blend ratio of spun yarn. An exponential equation to calculate tensile strength from an arbitrary UV exposure energy is evaluated for yarns and fabrics. The spun yarns were exposed to UV light using a xenon-arc weathering meter. The retention fraction of tensile strength decreased exponentially with increasing the fraction of UV exposure energy. Curve fitting of the retention fraction of tensile strength to the fraction of UV exposure energy revealed two groups of degradation coefficients based on the blending ratio of m-Aramid/p-Aramid. The correlation between the degradation coefficients ($\alpha_{y}$ and $\alpha_{f}$) of spun yarn and fabrics can be linearly regressed. The constant of proportionality in linear regression is considered to be the gap between the structure and the breaking mechanism of the fabric relative to yarn breakage. Based on the correlation between the degradation coefficients of spun yarn and fabrics and a mathematical model of the tensile strength of the spun yarn, the tensile strength of fabrics at a given UV exposure energy can be estimated from the tensile strength of the yarn.

## 1. Introduction

The combination of the mechanical properties of yarns and fabric structure is an essential element to consider for the mechanical properties of fabrics. Yarns used for protective clothing and industrial textiles are blended with various high-performance fibers for each application, and therefore, the degradation of their mechanical properties has numerous variations. Therefore, the degradation performance of fabrics needs fabric production and evaluation. As a result, there is the problem of time and cost. Furthermore, the relationship between the properties of the fiber, yarn properties (yarn count, structure), mechanical properties of the yarn (tensile, bending, compression), and fabric specifications (mass, structure) determines the tensile strength of the textile. Therefore, by showing these factors’ influence on the fabric’s tensile strength, it is possible to evaluate the mechanical properties of high-performance textile fabrics based on the properties of the yarns [1,2,3].

Researchers have analyzed the fracture mechanism of aramid fibers after UV exposure based on the fiber structure’s chemical structure, crystallinity, molecular weight, and surface features. Davis et al. [4] showed that the fracture mechanism of m-Aramid/p-Aramid fibers is the fragmentation of the aramid fiber surface into small fibrils, which propagate through the fiber by shear from the ends of the fibrils and finally fail. Aidani et al. [5] investigated the chemical structure and mechanical property changes of m-Aramid caused by UV irradiation. Infrared spectroscopy shows that the amide group (-NHCO-) of m-Aramid decomposes into carbonyl groups when exposed to UV light, based on the characteristic infrared absorption wavelength (1725 cm^−1^) of the carbonyl groups. Mechanical properties showed UV irradiation gave a decrease in tensile and tear strength. In addition, SEM observations showed that the formation of transverse cracks in the fiber after UV irradiation is due to fiber fracture, exfoliation that produces micropores, and longitudinal cracks in the fiber. The formulation of tensile strength relative to UV exposure energy is mainly for the degradation of a single fiber due to exposure to UV light.

Yamaguchi [6] reported that defects in the amorphous part of the fiber, where the bonding strength is weak, initiated the fracture of p-Aramid fibers, and tensile strength loss was correlated with the number of defects quantitatively. Based on the weakest link theory of Weibull [7], the tensile strength of p-Aramid fibers after UV irradiation can be quantitatively estimated. However, the study by Yamaguchi [6] modeled the reduction in tensile strength of p-Aramid fibers as a reinforcement material used in fiber-reinforced plastics under UV light, so the strength of m-Aramid fibers under UV light cannot be predicted from this study. Rezazadeh et al. [8] measured the residual strength of Nomex^®^ IIIA fabrics used in firefighter clothing when under heat exposure. This study developed a method for predicting residual strength using nondestructive testing in combination with near-infrared spectroscopic reflectance measurements of heat-exposed fabrics. The objective of this prediction method is to model tensile strength from 300 N to 600 N after thermal decomposition, in contrast to the numerical model of tensile strength loss to UV light. The numerical model uses the reflectance of the heat-exposed fabrics in three infrared wavelength ranges as variables. 

Wakatsuki et al. [9] found a quantitative relationship between UV exposure and strength retention for nine fabrics blended with m-Aramid and p-Aramid. This study also developed an equation to calculate tensile strength from arbitrary UV exposure energy and validated the replacement condition of firefighter clothing in NFPA 1851 [10] with a case study. The correlation between UV exposure energy and tensile strength in woven fabrics was qualitatively the same as that found for fibers by Yamaguchi [6]. Therefore, it is considered that defects resulting from changes in the aramid fiber interior commonly lead to a decrease in tensile strength for fibers, spun yarns, and woven fabrics.

Previous studies [4,5,6,9] evaluated the decrease in the tensile strength of fibers and fabrics after UV exposure. However, a correlation between the reduction of tensile strength of a fiber, a spun yarn, and a fabric as woven fiber assemblies after UV exposure was not investigated when the same fibers were used. Therefore, comparing the degradation coefficients of tensile strength of yarns and fabrics to UV light and establishing a prediction method that fills the gap will enable efficient design and degradation prediction of high-performance fabrics in the future at a low time and low cost.

This study aims to investigate and characterize UV exposure effect on the tensile strength of m-Aramid/p-Aramid blended yarns. Next, this study makes the curve-fitting formula for the retention fraction of tensile strength after UV exposure. Finally, a comparison was made with the model equations for tensile strength of yarns to woven fabrics made of m-Aramid/p-Aramid blended yarns of the exact specifications [9].

## 2. Experiment

### 2.1. Specimen Preparation

**Figure 1:**
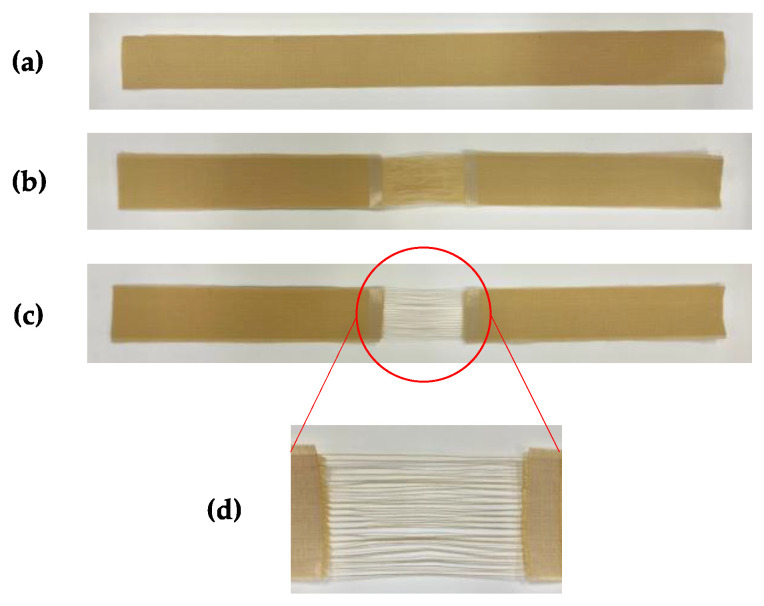
Preparation of yarn samples made from firefighter clothing outer layer fabrics (Sample I). (**a**–**d**) show the procedure for preparing yarn specimens for UV exposure, respectively: (**a**) a woven fabric for preparing yarn specimens, (**b**) unraveled weft yarn, (**c**) thinning out of warp yarn, and (**d**) enlargement of the red circle in (**c**).

**Figure 2:**
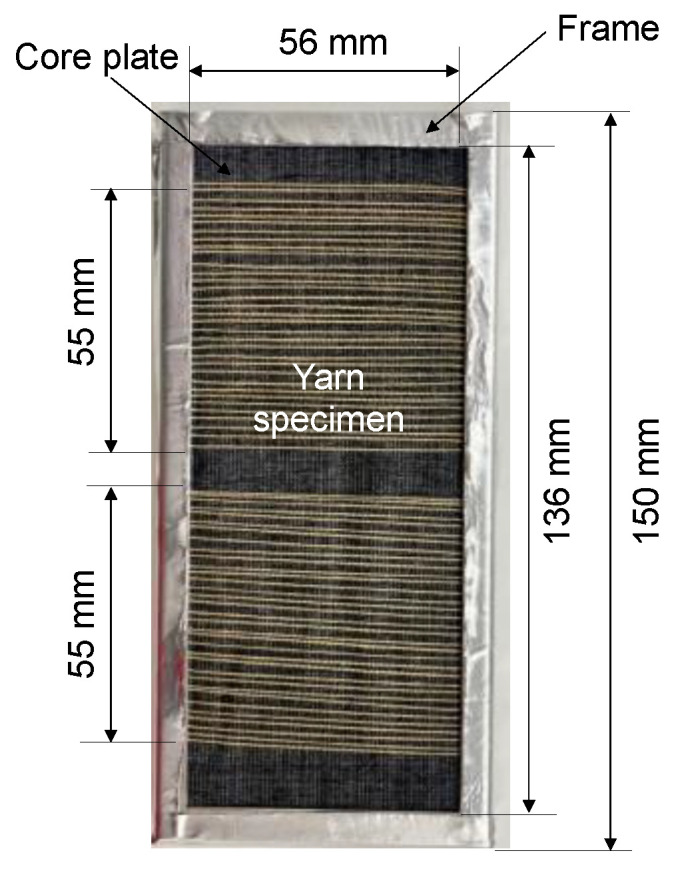
Yarn specimen placement for UV exposure in the test frame.

The spun yarns for UV exposure were two-ply spun yarns in warp direction sampled from fabrics used in the tensile test of firefighter clothing outer layers [9]. Table 1 shows the specification of yarn samples for tests. The yarns were made of 100% m-Aramid, 100% p-Aramid, and a blend of m-Aramid and p-Aramid. Figure 1 shows the procedure for fabricating a 500 mm long spun yarn specimen. Since the UV exposure conditions shall be the same as those of the three-layer fabric of firefighter clothing, the yarn specimens were layered in the order of yarn specimen, moisture barrier, and thermal liner from the top, as shown in Figure 2. Specimen preparation is consistent with the one for fabrics [9].

The surface area of warp yarns exposed to UV light depends on the spacing between the warp yarns, and the tensile strength of the warp yarns changes with the surface area. When exposed to ultraviolet light, the tensile strength of the warp yarns with wide spacing between the warp yarns is lower than that of the warp yarns with narrow spacing. Therefore, the method of unraveling warp yarns was investigated in preparing the UV exposure specimens. It was found that when the warp yarn spacing was wider than every five warp yarns, i.e., when four warp yarns were unraveled from five warp yarns, one warp yarn was left, and the tensile strength remained constant. Therefore, all specimens used in this study for UV exposure were prepared under these conditions. 

Figure 1c,d show specimens of the yarn for UV exposure. The UV exposure specimens were set on the test frame, as shown in Figure 2. The warp yarn specimens used in the tensile test were made by trimming 50 mm from both ends of a 600 mm long specimen used in the UV exposure test, then the weft yarn was unraveled for a length of 500 mm.

### 2.2. UV Exposure Methods and Conditions

The UV exposure method was the same as in [9] for comparing the degradation coefficient of tensile strength of spun yarn and woven fabrics to UV light. A xenon-arc weathering meter (SX 75, Suga test instrument) with radiation intensity at 180 W/m^2^ was used to prepare the UV-exposed yarn specimen. Following ISO 4982-2 [11], the temperature and relative humidity in the weather meter chamber were 63 °C and 50% RH, respectively. The UV exposure in this study focused on the degradation caused by UV exposure only, without the injection of distilled water to reproduce actual weather conditions. 

The test frames with the yarn specimens attached were periodically exchanged between the top, middle, and bottom positions to minimize variation in UV exposure conditions. The area of the UV exposure was 55 mm in the weft direction and 28 mm up and down from the fabric center in the warp direction (56 mm in total). 

**Figure 3:**
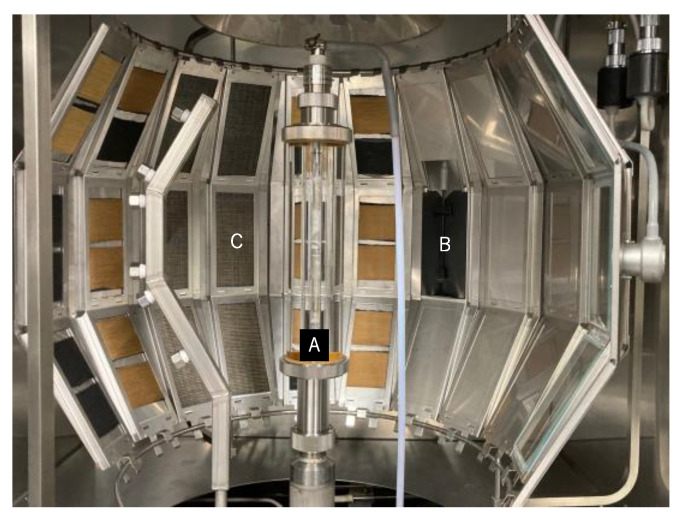
Example of test frame placement in Xenon-arc weathering meter. The letters “A”, “B”, and “C” represent the xenon-arc lamp, the black panel thermometer, and the test frame with two yarn specimens, respectively.

Figure 3 shows the test frame mounted in a sample holder in the chamber of the xenon-arc weathering meter. The letters A, B, and C in Figure 3 represent a specimen rack with a xenon arc lamp, a black panel thermometer, and two yarn specimens for exposure, respectively. Conditions of UV exposure shown in Table 2 were determined based on ISO 4892-2 [11], JIS D 0205:1987 [12], and the annual average of 12.08 h [13] of direct solar radiation per day in Japan. Based on these assumptions, the UV exposure energy at the wavelengths was determined to be 34 MJ/m^2^ [9]. The UV exposure time was determined using the output of a xenon arc lamp, based on Equation (1). The UV exposure energy of 340 MJ/m^2^ assumes that firefighter clothing will be used for ten years [9].

(1) $$Exposure\;time\;in\;hours\;per\;year=\frac{UV\;exposure\;energy\;per\;year\;}{Radiation\;intensity\;of\;xenon\;arc\;lamp}$$

### 2.3. Tensile Strength Test

The tensile test of spun yarn was conducted according to JIS L 1095:2010 [14] with a tensile tester (RTC-1250A, A&D). Two load cells, a 500 N load cell for unexposed yarn specimens, and a 50 N load cell for UV-exposed yarn specimens, were used for the tensile test. The tensile strength test was conducted at a speed of 125 mm/min, a distance between chucks of 250 mm, and a sampling period of 0.01 s. The specimens were clamped with a yarn tensile chuck (A&D, J-JTA-500 N/2.5 kN). Twenty times for each exposure energy for each sample were examined. The tensile strength in this study was the average value of the maximum tensile strength (cN). Equation (2) is the retention fraction of tensile strength introduced as the fraction of tensile strength of the exposed specimen to that of the unexposed specimen.

(2) $$\begin{array}{c} Retention\;fraction\;of\;tensile\;strength=\frac{Tensile\;strength\;of\;the\;exposed\;specimen\;}{Tensile\;strength\;of\;the\;unexposed\;specimen} \end{array}$$

## 3. Results

Table 3 shows the tensile strength and retention fraction of tensile strength (*I/I*_0_) of spun yarn blended with m-Aramid and p-Aramid. The relationship of the tensile strength (*I*) to UV exposure energy (*Q*) and retention fraction of tensile strength (*I/I*_0_) to the fraction of UV exposure energy (*Q/Q*_0_) were analyzed. The fraction of UV exposure energy corresponding to the number of years of exposure (*Q/Q*_0_) is the fraction of UV exposure energy (*Q*) to UV exposure energy per year (*Q*_0_ = 34 MJ/m^2^).

### 3.1. Effect of Yarn Size on Tensile Strength

**Figure 4:**
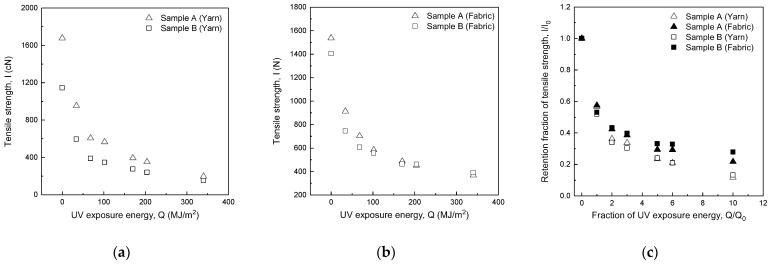
Change in tensile strength of yarns (**a**) and fabrics (**b**) [9] of two different yarn diameters (100% m-Aramid) and retention fraction of tensile strength (**c**) [9].

Figure 4a and Table 4 show the tensile strength (*I*) of yarn as a function of UV exposure energy (*Q*). As shown in Table 1, samples A and B have the same fiber and yarn structure (100% m-Aramid, two-ply) but different yarn sizes (yarn counts). The difference in tensile strength between samples A and B decreased with increasing UV exposure energy. The average percentage difference in tensile strength at UV exposure energy *Q* = 34–102 MJ/m^2^ was 59.5%, and that at UV exposure energy *Q* = 170–340 MJ/m^2^ was 39.7%. Although the difference in the percentage of tensile strength at UV exposure energy *Q* = 170–340 MJ/m^2^ was smaller than that at UV exposure energy *Q* = 34–102 MJ/m^2^, the difference was clearly due to the dependence of tensile strength on yarn size. 

Figure 4a,b show a comparison of the tensile strength (*I*) of yarns and fabrics of samples A and B against UV exposure energy (*Q*). The tensile strength (*I*) of yarns and fabrics decreases exponentially with UV exposure energy (*Q*). However, the tensile strength of yarns of samples A and B is affected by the yarn size, while the effect of yarn size is not significant for the woven fabrics for UV exposure energies above *Q* = 102 MJ/m^2^. 

Figure 4c shows the fraction of UV exposure energy (*Q*/*Q*_0_) and retention fraction of tensile strength (*I*/*I*_0_) for sample A and B yarns and fabrics. As shown in Table 4, the difference in the retention fraction of tensile strength between samples A and B was as small as ±0.05. Therefore, the retention fraction of tensile strength (*I*/*I*_0_) to the fraction of UV exposure energy (*Q*/*Q*_0_) is independent of yarn size. The retention fraction of tensile strength (*I*/*I*_0_) was the same for yarns and fabrics up to the fraction of UV exposure energy of *Q*/*Q*_0_ = 1. The retention fraction of yarns and fabrics’ tensile strength (*I*/*I*_0_) differed when *Q*/*Q*_0_ exceeded 2, and the difference in *I*/*I*_0_ increased as the fraction of UV exposure energy (*Q*/*Q*_0_) increased. The retention fraction of fabrics’ tensile strength (*I*/*I*_0_) was always higher than that of yarn.

### 3.2. Effect of Blending Ratio of m-Aramid/p-Aramid on Tensile Strength and Retention Fraction of Tensile Strength

**Figure 5:**
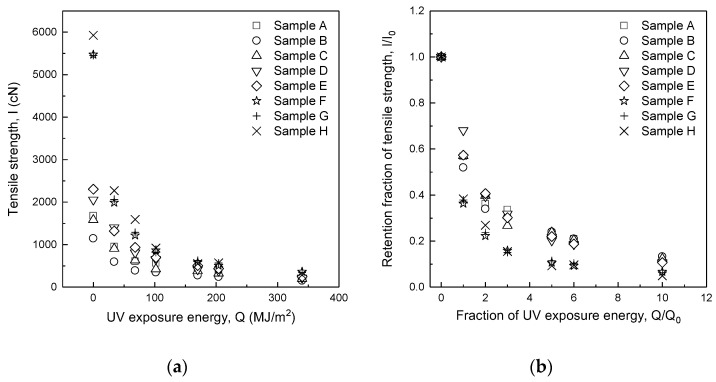

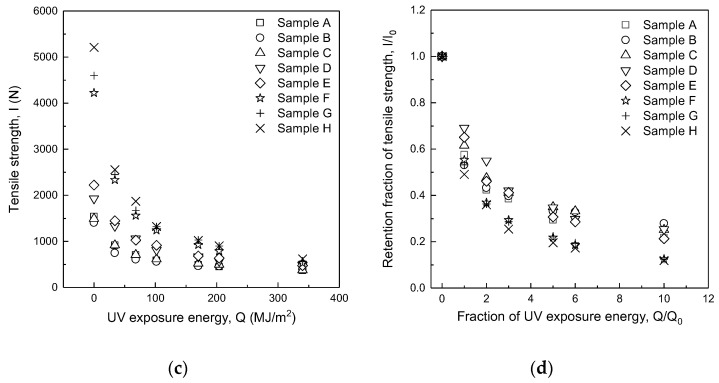
Comparison of yarn tensile strength (**a**) and retention fraction of tensile strength (**b**) and fabric data (**c**,**d**) for different m-Aramid/p-Aramid blends.

Figure 5a,c and Table 3 show the tensile strength (*I*) as a function of UV exposure energy (*Q*) for the yarns and fabrics of all samples. In common with all samples, tensile strength (*I*) as a function of UV exposure energy (*Q*) decreased significantly and exponentially up to a UV exposure energy of *Q* = 102 MJ/m^2^ and then decreased slowly. This characteristic is consistent with the fabrics’ tensile strength (*I*) to UV exposure energy (*Q*). The role of p-Aramid in providing higher tensile strength decreases with increasing UV exposure energy (*Q*). 

Figure 5b,d and Table 3 show the relationship between the fraction of UV exposure energy (*Q/Q*_0_) and the retention fraction of tensile strength (*I*/*I*_0_) for the yarns and fabrics of all samples. The retention fraction of tensile strength (*I*/*I*_0_) of the yarns and that of the fabrics decreased significantly and exponentially up to the fraction of UV exposure energy of *Q/Q*_0_ = 3 and then decreased slowly. The retention fraction of tensile strength (*I*/*I*_0_) of yarns was lower than that of fabrics at the same fraction of UV exposure energy (*Q/Q*_0_), indicating that the retention fraction of tensile strength (*I*/*I*_0_) of yarns decreased faster than that of fabrics.

When the fraction of UV exposure energy of *Q*/*Q*_0_ = 1, i.e., one year of firefighter clothing use, the retention fraction of tensile strength (*I*/*I*_0_) was lower than 0.5 for samples F, G, and H in which the p-Aramid blend ratio was high. However, the retention fraction of tensile strength (*I*/*I*_0_) of the fabrics of the same samples, F, G, and H, was greater than 0.5.

As with the tensile strength results in Figure 4, the difference in the retention fraction of tensile strength (*I*/*I*_0_) between yarns and fabrics can be attributed to the following three points, regardless of the blend ratio of m-Aramid and p-Aramid. First, yarns have a larger surface area exposed to UV radiation than woven fabrics. The depth at which UV radiation reaches the interior of yarns differs between the case of yarns alone and woven fabrics. Second, the intersection of warp and weft yarns in woven fabrics is structurally affected by UV exposure. Third, the breakage of woven fabrics during tension is not only by the breakage of yarns but also by the breakage of the weft yarns when the fabrics are drawn. Finally, the breakage of the fabric in tension is caused by the combined resistance forces of bending and compression that occur at the intersection of warp and weft yarns.

The retention fraction of tensile strength (*I*/*I*_0_) for all yarns and fabrics is between sample A, 100% m-Aramid, and sample H, 100% p-Aramid. Therefore, the retention fraction of tensile strength (*I*/*I*_0_) of yarns blended with m-Aramid and p-Aramid can be estimated with the same approach as woven fabrics.

### 3.3. Curve Fitting of Retention Fraction of Tensile Strength

**Figure 6:**
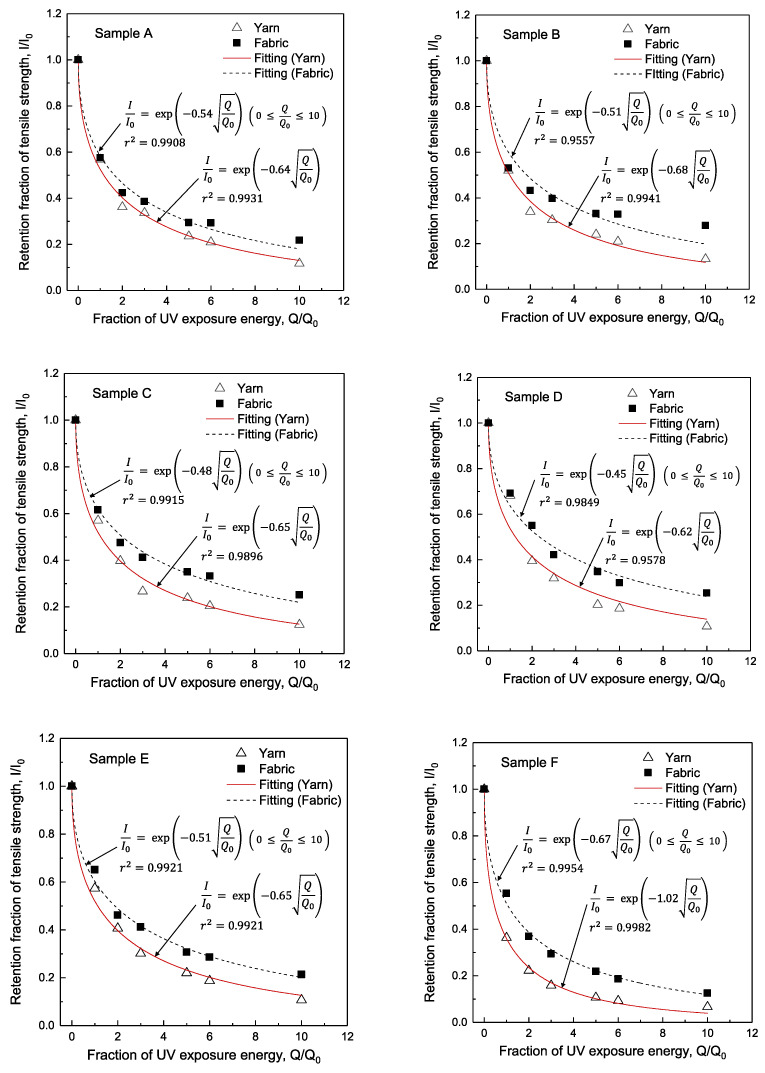

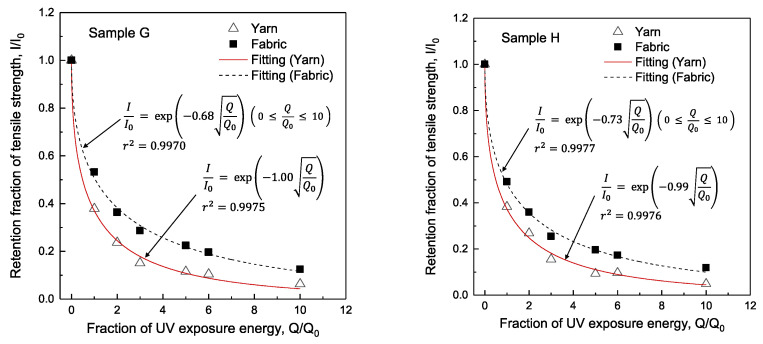
Relationship between the fraction of UV exposure energy and retention fraction of tensile strength of yarns and fabrics on Samples A to H. White- and black-filled symbols represent experimental data of yarn and fabric, respectively. The solid and dotted lines represent the fitting data of the yarn and fabric by Equation (3).

Figure 6 shows the relationship between the fraction of UV exposure energy (*Q*/*Q*_0_) and the retention fraction of tensile strength (*I*/*I*_0_) for all samples. Each graph includes the curve-fitting results. In addition, Figure 6 contains the fitting results for woven fabrics. The solid red line in each graph represents the fitting results for yarn, and the black dotted line represents the fitting results for woven fabrics.

Equation (3) is the equation for curve-fitting between the fraction of UV exposure energy (*Q*/*Q*_0_) and the retention fraction of tensile strength (*I*/*I*_0_) [9]. The retention fraction of tensile strength (*I*/*I*_0_) decreases exponentially with increasing the fraction of UV exposure energy (*Q*/*Q*_0_) and can be expressed as $\sqrt{Q/Q_{0}}$. Therefore, due to UV exposure, a higher degradation coefficient of yarn ($\alpha_{y}$) is the rapid loss of tensile strength.

(3) $$\begin{array}{c} \;\frac{I}{I_{0}}=exp\left(-\alpha_{y}\sqrt{\frac{Q}{Q_{0}}}\right)\;\left(\;0\;\leq \;\frac{Q}{Q_{0}}\;\leq \;10\;\right) \end{array}$$

**Figure 7:**
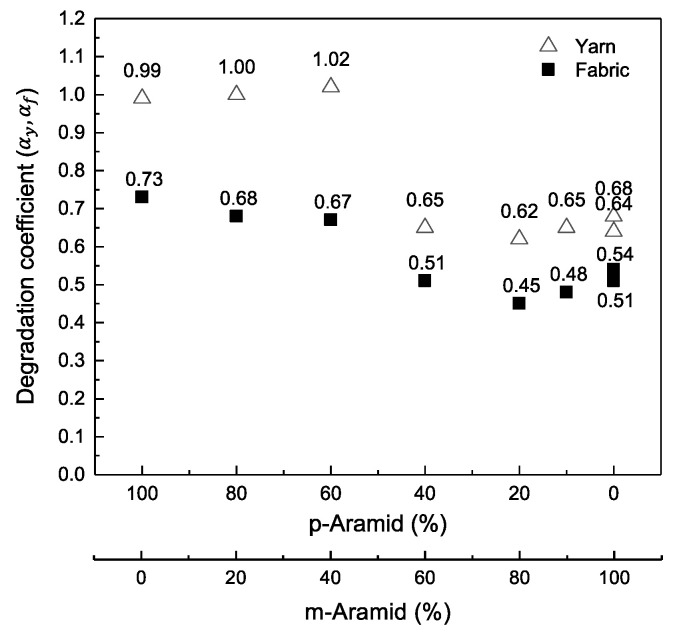
Relationship between degradation coefficient of yarn and fabric ($\alpha_{y}$ and $\alpha_{f}$) by the blend of m-Aramid/p-Aramid based on Equation (3).

Figure 7 and Table 5 show a comparison of the degradation coefficients $\alpha_{y}$ and $\alpha_{f}$ [9] of yarns and fabrics for m-Aramid and p-Aramid blends, respectively. The degradation coefficients ($\alpha_{y}$) for all samples in this study range from 0.64 and 0.99 for sample A (m-Aramid 100%) and sample H (p-Aramid 100%), respectively.

The yarn degradation coefficient ($\alpha_{y}$) was divided into two groups: more than 60% of m-Aramid blends and less than 40% of m-Aramid blends. When m-Aramid blends were 60% or more, the average degradation coefficient ($\alpha_{y}$) for aramid blended fabrics with an emphasis on thermal resistance was 0.65. The average degradation coefficient ($\alpha_{y}$) for yarns blended with 40% or less m-Aramid, i.e., for blended yarns with emphasis on strength, was 1.00. The degradation factor ($\alpha_{y}$) for yarns was higher than that for fabrics ($\alpha_{f}$) reported previously [9]. In other words, the tensile strength of UV-exposed yarns qualitatively degrades faster than that of woven fabrics.

Spun yarns produce strength when the fibers tighten to the central axis of the yarn in tensile strength. If the spun yarn has a high p-Aramid fiber blend, when the p-Aramid fiber breaks, the m-Aramid fiber with a low blend ratio breaks because it cannot withstand the strength. As a result, the degradation factor ($\alpha_{y}$) is close to the 100% p-Aramid fiber value.

In contrast, in the case of spun yarn with a high blend ratio of m-Aramid fibers, the p-Aramid fibers, which deteriorate rapidly under UV light, may break. Still, the m-Aramid fibers, which deteriorate slowly under UV light, may not break due to the low blend ratio of p-Aramid fibers. Consequently, the degradation coefficient ($\alpha_{y}$) is close to the 100% m-Aramid fiber value. 

The yarns of Sample B (m-Aramid 100%) and Sample H (p-Aramid 100%) have the same structure and yarn size. However, comparing the fraction of UV exposure energy (*Q*/*Q*_0_) and the retention fraction of tensile strength (*I*/*I*_0_) for the two yarns shown in Figure 6, the degradation factor ($\alpha_{y}$) is higher for Sample H than for Sample B. This result indicates that p-Aramid yarn loses tensile strength faster than m-Aramid yarn due to UV exposure. 

The degradation factor ($\alpha_{y}$) is related to the number of defects in the aramid fiber caused by UV exposure [6], and the degree of reduction in tensile strength contributed by each defect. For example, suppose the number of defects in the m-Aramid and p-Aramid fibers caused by UV exposure is the same. In that case, the effect of one defect on the reduction in tensile strength of the m-Aramid and p-Aramid fibers is different. On the other hand, if the effect of a single defect in the fiber on the tensile strength reduction by UV exposure is the same for m-Aramid and p-Aramid fibers, the number of defects in the fiber by UV exposure would be different. Therefore, it is important to understand tensile strength loss characteristics by investigating the number of defects in aramid fibers. Additionally, due to UV exposure, tensile strength is lost to quantitatively characterize the tensile strength of p-Aramid and m-Aramid blended yarns to UV exposure energy.

**Figure 8:**
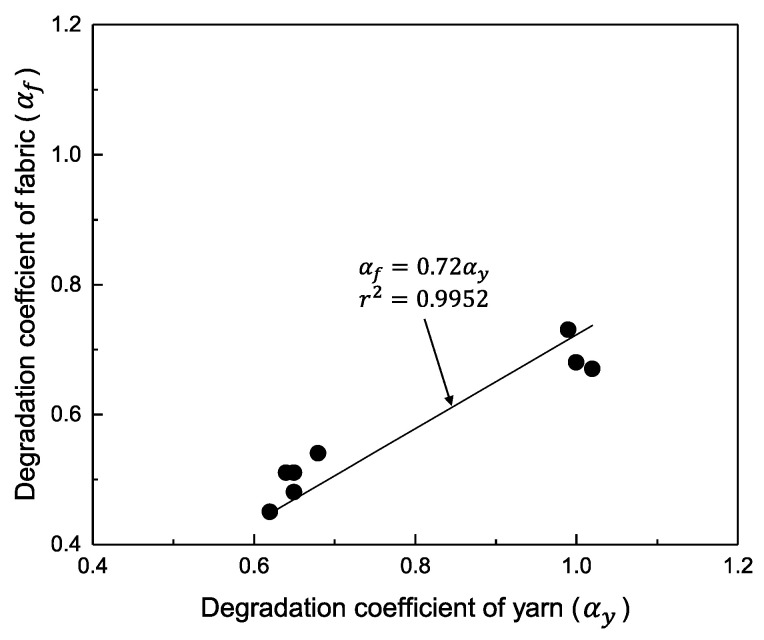
Relationship between degradation coefficient of yarn and fabric ($\alpha_{y}$ and $\alpha_{f}$) by the blend of m-Aramid/p-Aramid based on Equation (3).

Figure 8 shows the results of the correlation and linear regression of the degradation coefficients ($\alpha_{y}$ and $\alpha_{f}$) for spun yarn and woven fabrics. The degradation coefficients ($\alpha_{y}$ and $\alpha_{f}$) of spun yarn and woven fabrics are proportional, with a slope of 0.72 and a correlation coefficient *r^2^* of 0.9952. Therefore, the estimation of the tensile strength of fabrics from yarns to fabrics in this study can be made by applying the coefficients obtained from the linear regression shown in Figure 8 as correction parameters to the degradation coefficients of the tensile strength of yarns. The results indicate that it is possible to predict the tensile strength of UV-exposed fabrics by examining the tensile strength of yarns to UV exposure.

## 4. Conclusions

The dependence of blending ratio, yarn thickness, and UV exposure energy (*Q*) on tensile strength (*I*) by UV degradation was investigated for spun yarns for outer layer fabrics of firefighter clothing. UV exposure of spun yarns with eight types of the m-Aramid/p-Aramid blend was conducted by a xenon-arc weathering meter. Characteristics of tensile strength of UV exposure showed that the retention fraction of tensile strength by UV exposure depended on blending m-Aramid and p-Aramid fibers. In addition, all blends’ tensile strength (*I*) was exponentially decayed with UV exposure energy (*Q*). Tensile strength change for all samples is within the result of 100% m-Aramid, and 100% p-Aramid spun yarns. In addition, the yarn size dependence of tensile strength was observed for all UV exposure energies but not for the retention fraction of tensile strength (*I/I*_0_).

For all spun yarns, the retention fraction of tensile strength (*I*/*I*_0_) decreased exponentially with an increasing fraction of UV exposure energy (*Q*/*Q*_0_). Therefore, by fitting the retention fraction of tensile strength (*I*/*I*_0_) to the fraction of UV exposure energy (*Q*/*Q*_0_), the degradation coefficients ($\alpha_{y}$) for all yarns were almost within the value of 0.64 and 0.99 for sample A (m-Aramid 100%) and sample H (p-Aramid 100%), respectively. 

The degradation coefficient for yarn ($\alpha_{y}$) was always higher than that for fabric ($\alpha_{f}$). In other words, the tensile strength of yarns degrades qualitatively faster than that of woven fabrics due to UV exposure. This difference is attributed to a surface area and the depth at which UV radiation was exposed, exposure conditions, and the combined resistance forces of bending and compression at the intersection of warp and weft yarns in woven fabrics. Furthermore, the correlation between the degradation coefficients ($\alpha_{y}$ and $\alpha_{f}$) of spun yarn and woven fabrics can be linearly regressed. Therefore, the prediction of the tensile strength of woven fabrics under UV exposure is possible by applying the coefficients from linear regression to the degradation model of spun yarn under UV exposure used in this study. The results indicate that it is possible to estimate the tensile strength of UV-exposed fabrics by examining the tensile strength of yarns to UV exposure.

## Figures and Tables

**Table 1:** Specification of the test yarn.

Sample	Fiber and Blending Ratio (%)	Yarn Count, Ne (dtex)
A	m-Aramid = 100	18.9 (312)
B	m-Aramid = 100	25.0 (236)
C	m-Aramid/p-Aramid = 90/10	20.0 (295)
D	m-Aramid/p-Aramid = 80/20	18.9 (312)
E	m-Aramid/p-Aramid = 60/40	18.9 (312)
F	m-Aramid/p-Aramid = 40/60	18.9 (312)
G	m-Aramid/p-Aramid = 20/80	18.9 (312)
H	p-Aramid = 100	25.0 (236)

**Table 2:** Conditions to prepare UV-exposed yarn specimens.

Estimated Years (Year)	0	1	2	3	5	6	10
UV exposure energy (MJ/m^2^)	0	34	68	102	170	204	340
Exposure time (hour)	0	52.4	104.8	157.2	262.0	314.4	524.0

**Table 3:** Tensile strength and the retention fraction of tensile strength for m-Aramid/p-Aramid blending yarns.

		UV Exposure Dosage (MJ/m^2^)						
	*Q* (MJ/m^2^)	0	34	68	102	170	204	340
	*Q*/*Q_0_*	0	1	2	3	5	6	10
A	*I* (cN)	1677.2	951.9	606.8	564.6	394.1	351.4	196.3
	*s*(cN)	132.2	67.7	52.7	62.2	44.9	57.7	45.1
	*I*/*I*_0_	1.00	0.57	0.36	0.34	0.23	0.21	0.12
B	*I* (cN)	1145.0	594.5	389.0	347.4	275.2	239.7	151.9
	*s* (cN)	73.7	48.8	38.1	20.3	36.5	29.6	26.1
	*I*/*I_0_*	1.00	0.52	0.34	0.30	0.24	0.21	0.13
C	*I* (cN)	1583.6	903.3	629.2	422.1	377.0	323.7	195.7
	*s* (cN)	88.6	89.4	53.9	36.9	50.4	62.1	22.2
	*I*/*I*_0_	1.00	0.57	0.40	0.27	0.24	0.20	0.12
D	*I* (cN)	2058.1	1402.5	811.5	656.3	417.3	383.2	221.1
	*s* (cN)	175.4	87.1	108.5	93.5	85.9	76.2	46.8
	*I*/*I*_0_	1.00	0.68	0.39	0.32	0.20	0.19	0.11
E	*I* (cN)	2303.2	1320.8	935.5	694.7	506.0	431.8	245.0
	*s* (cN)	148.4	105.5	69.1	55.6	67.1	38.7	36.7
	*I*/*I*_0_	1.00	0.57	0.41	0.30	0.22	0.19	0.11
F	*I* (cN)	5472.1	1986.3	1215.9	867.9	582.6	505.8	363.2
	*s* (cN)	580.0	156.5	116.7	86.6	90.8	59.5	57.3
	*I*/*I*_0_	1.00	0.36	0.22	0.16	0.11	0.09	0.07
G	*I* (cN)	5449.4	2063.8	1290.6	819.8	627.6	566.9	343.7
	*s* (cN)	408.5	187.2	119.9	80.5	75.1	67.4	32.4
	*I*/*I*_0_	1.00	0.38	0.24	0.15	0.12	0.10	0.06
H	*I* (cN)	5925.8	2268.3	1592.4	917.8	548.8	572.2	289.1
	*s* (cN)	613.8	229.6	184.8	111.2	59.9	94.8	46.5
	*I*/*I*_0_	1.00	0.38	0.27	0.15	0.09	0.10	0.05

**Table 4:** Effect of yarn size on tensile strength and retention fraction of tensile strength (Samples A and B).

		UV Exposure Dosage (MJ/m^2^)						
	*Q* (MJ/m^2^)	0	34	68	102	170	204	340
	*Q*/*Q_0_*	0	1	2	3	5	6	10
A	*I* (cN)	1677.2	951.9	606.8	564.6	394.1	351.4	196.3
	*I*/*I*_0_	1.00	0.57	0.36	0.34	0.23	0.21	0.12
B	*I* (cN)	1145.0	594.5	389.0	347.4	275.2	239.7	151.9
	*I*/*I*_0_	1.00	0.52	0.34	0.30	0.24	0.21	0.13
$I_{A}-I_{B}$	(cN)	532.2	357.4	217.8	217.2	118.9	111.7	44.4
$\frac{I_{A}-I_{B}}{I_{B}}$	(%)	46.5	60.1	56.0	62.5	43.2	46.6	29.2
${\left[I/I_{0}\right]}_{A}-{\left[I/I_{0}\right]}_{B}$		0.00	0.05	0.02	0.04	−0.01	0.00	−0.01

**Table 5:** Difference of degradation coefficients of yarn and fabric ($\alpha_{y}$ and $\alpha_{f}$) by the blend of m-Aramid/p-Aramid.

Sample	A	B	C	D	E	F	G	H
m-Aramid (%)	100	100	90	80	60	40	20	0
p-Aramid (%)	0	0	10	20	40	60	80	100
$\alpha_{y}$	0.64	0.68	0.65	0.62	0.65	1.02	1.00	0.99
$\alpha_{f}$	0.54	0.51	0.48	0.45	0.51	0.67	0.68	0.73
$\alpha_{y}-\alpha_{f}$	0.10	0.17	0.17	0.17	0.14	0.35	0.32	0.26
$\frac{\alpha_{y}-\alpha_{f}}{\alpha_{f}}\;()$	18.5	33.3	35.4	37.8	27.5	52.2	47.1	35.6
